# Streamlined production, purification, and characterization of recombinant extracellular polyhydroxybutyrate depolymerases

**DOI:** 10.1002/mbo3.1001

**Published:** 2020-02-22

**Authors:** Diana I. Martínez‐Tobón, Brennan Waters, Anastasia L. Elias, Dominic Sauvageau

**Affiliations:** ^1^ Department of Chemical and Materials Engineering University of Alberta Edmonton AB Canada

**Keywords:** *Escherichia coli* vectors, extracellular PHB depolymerases (PhaZs), poly(3‐hydroxybutyrate) (PHB), polymer degradation activity, recombinant expression

## Abstract

Heterologous production of extracellular polyhydroxybutyrate (PHB) depolymerases (PhaZs) has been of interest for over 30 years, but implementation is sometimes difficult and can limit the scope of research. With the constant development of tools to improve recombinant protein production in *Escherichia coli*, we propose a method that takes characteristics of PhaZs from different bacterial strains into account. Recombinant His‐tagged versions of PhaZs (rPhaZ) from *Comamonas testosteroni* 31A, *Cupriavidus* sp. T1*, Marinobacter algicola* DG893, *Pseudomonas stutzeri*, and *Ralstonia* sp. were successfully produced with varying expression, solubility, and purity levels. PhaZs from *C. testosteroni* and *P. stutzeri* were more amenable to heterologous expression in all aspects; however, using the *E. coli* Rosetta‐gami B(DE3) expression strain and establishing optimal conditions for expression and purification (variation of IPTG concentration and use of size exclusion columns) helped circumvent low expression and purity for the other PhaZs. Degradation activity of the rPhaZs was compared using a simple PHB plate‐based method, adapted to test for various pH and temperatures. rPhaZ from *M. algicola* presented the highest activity at 15°C, and rPhaZs from *Cupriavidus* sp. T1 and *Ralstonia* sp. had the highest activity at pH 5.4. The methods proposed herein can be used to test the production of soluble recombinant PhaZs and to perform preliminary evaluation for applications that require PHB degradation.

## Graphical abstract

A rapid, efficient approach for the expression of active recombinant extracellular polyhydroxybutyrate (PHB) depolymerases originating from various bacterial strains. It enables the characterization of new PHB depolymerases and the direct comparison of activity between enzymes and conditions; as an example, the activity of five PHB depolymerases is evaluated and compared at different pH and temperatures. This approach can accelerate the investigation and development of applications based on the degradation of biopolymers.

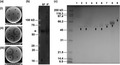

## 1 INTRODUCTION

The study of extracellular polyhydroxybutyrate (PHB) depolymerases (PhaZs) produced by a variety of microorganisms (Jendrossek, 2005; Knoll et al., 2009; Roohi et al., 2017) remains an important and evolving research area. Their enzymatic activity results in the degradation of PHB, a natural biodegradable polymer with the potential to replace some currently widely used petroleum‐based plastics (Volova, 2004) that increasingly accumulate in the environment (Geyer, Jambeck, & Law, 2017).

Recombinant protein production is a powerful tool that allows the production of higher levels of proteins in expression systems such as *Escherichia coli*. Optimized recombinant technologies facilitate purification, the study of proteins in isolation, the conception of a platform to modify and improve them, and the development of new applications. In the case of PhaZs, such applications include biosensors—such as time–temperature indicators (Anbukarasu et al., 2017) and pathogen detection platforms (Elias et al., 2018)—and recycling of biodegradable polymers (Lee et al., 2018).

Examples of expression of rPhaZs in *E. coli* include PhaZ2–PhaZ3 (Briese, Schmidt, & Jendrossek, 1994) and PhaZ7 (although for this specific PhaZ better expression was achieved with in *Bacillus subtilis* WB800) (Braaz, Handrick, & Jendrossek, 2003) from *Paucimonas lemoignei* and PhaZ from *Caldimonas manganoxidans* (Takeda et al., 2000; Lee et al., 2018). In some cases, purification of rPhaZs has also been performed: several PhaZs from *P. lemoignei* (PhaZ1–PhaZ5 (Jendrossek, Frisse, et al., 1995; Jendrossek, Müller, & Schlegel, 1993), PhaZ7 and related mutants (Jendrossek, Hermawan, Subedi, & Papageorgiou, 2013)), *Pseudomonas stuzeri* (Ohura, Kasuya, & Doi, 1999), *Alcaligenes faecalis* AE122 (Kita et al., 1997), *Marinobacter* sp. NK‐1 (Kasuya et al., 2003), *Bacillus megaterium* N18‐25‐9 (Takaku, Kimoto, Kodaira, Nashimoto, & Takagi, 2006), *Pseudomonas mendocina* DSWY0601 (Wang et al., 2012), and from *Cupriavidus* sp. T1 (formerly *Alcaligenes faecalis* T1 (Oshida, Kitamura, Iida, & Ohkuma, 2015; JCM n.d.)) and related mutants (Hiraishi, Hirahara, Doi, Maeda, & Taguchi, 2006; Hiraishi et al., 2009; Saito et al., 1989; Tan, Hiraishi, Sudesh, & Maeda, 2013, 2014). However, these studies each required the development of specific methods for heterologous expression of specific PhaZs. In addition, in many cases affinity tags were not employed, requiring significant additional steps for purification (Hiraishi et al., 2006, 2009; Jendrossek, Frisse, et al., 1995; Jendrossek et al., 1993; Kita et al., 1997; Saito et al., 1989). These factors impede on the rapidity and scope of studies, even limiting comparisons between PhaZs.

In this study, we established a platform for the rapid expression and purification of extracellular rPhaZs. This was demonstrated with five extracellular PhaZs displaying different properties and of various bacterial origins. Predicted solubility and disulfide bonds (necessary for maintaining proper conformation and activity in many proteins (Rosano & Ceccarelli, 2014)) of the rPhaZs produced were important criteria in selecting the *E. coli* system, specifically the plasmid vector and expression strains. Although *E. coli* is not recognized as an ideal expression host for the production of extracellular proteins, specific *E. coli* strains, such as *E. coli* Rosetta‐gami B(DE3), can have clear advantages for the production of rPhaZ. A single platform with simple strategies was successfully employed for expression, purification, and preliminary comparison of degradation performance under different conditions.

## 2 MATERIALS AND METHODS

### 2.1 Bacterial strains and growth conditions

The bacterial strains used for isolation of the PhaZs, cloning and expression, as well as their growth medium and conditions, can be found in Table 1. Cell growth was monitored by measuring optical density of the cultures at 600 nm (OD_600_) using a UV‐Vis spectrophotometer (Biochrom, Ultrospec 50). Plating was performed on 1.5% w/v agar supplemented with the medium of interest and plates were incubated in a temperature‐controlled incubator (Isotemp 500 Series, Fisher Scientific).

**Table 1:** Bacterial strains, conditions, and primers. Growth conditions and information of (a) PhaZ‐producing strains and (b) cloning and expression strains; (c) rPhaZs primers

a. PhaZ‐producing strains information			
Strain, PhaZ name (gene)	Sampling environment	Growth conditions	Source and identification
*Comamonas testosteroni* 31A, PhaZ*_Cte_* (*phaZ_Cte_*)	Soil from a greenhouse	Tryptic soy broth, 30°C	DSMZ 6,781
*Cupriavidus* sp. T1, PhaZ*_C_*_sp_ (*phaZ_Csp_*)	Activated sludge obtained from the Toba sewage‐treatment plant, Kyoto, Japan	Nutrient broth, 30°C	Japan Collection of Microorganisms (JCM) 10,169
*Marinobacter algicola* DG893, PhaZ*_Mal_* (*phaZ_Mal_*)	Laboratory culture of dinoflagellate *Gymnodinium catenatum* YC499B15	Marine broth, 28°C	DSMZ 16,394
*Pseudomonas stutzeri*, PhaZ*_Pst_* (*phaZ_Pst_*)	Seawater, Jogashima, Kanagawa Pref., Japan	Nutrient broth, 30°C	JCM 10,168
*Ralstonia* sp., PhaZ*_R_*_sp_ (*phaZ_Rsp_*)	Atmosphere in the laboratory, Japan	Nutrient broth, 30°C	JCM 10,171

b. Cloning and expression strains		
Strain	Growth conditions	Source
NEB 5‐alpha Competent *E. coli* (Subcloning Efficiency)	Luria–Bertani (LB) media supplemented with ampicillin or carbenicillin (100 µg/ml), 37°C, 250 rpm	New England Biolabs (NEB)
*E. coli* ElectroMAX DH10B	LB media supplemented with ampicillin or carbenicillin (100 µg/ml), 37°C, 250 rpm	ThermoFisher Scientific
*E. coli* Rosetta‐gami B(DE3)	LB media in the presence of chloramphenicol (34 µg/ml), kanamycin (15 µg/ml), tetracycline (12.5 µg/ml), and carbenicillin (50 µg/ml), 30 or 32°C for precultures, 37°C and 15°C overnight during induction, 250 rpm. For plate cultures incubation was done for at least 24 hr	Novagen
T7 Express *lysY/I^q^ E. coli*	LB media supplemented with ampicillin or carbenicillin (100 µg/ml), 30 or 32°C for precultures, 37°C and 15°C overnight during induction, 250 rpm	NEB

c. Primers used to produce mature rPhaZs		
*phaZ* gene	Primer number	5' to 3' sequence
*phaZ_Cte_*	1	CAATTTACGACA**GAATTC**CGCCGTGCCGCTGGGGCAATACAACATT
	2	GCGATAAACAAT**CTCGAG**GGGGCAGGTACCGATCACGTAGTAGTTGCT
*phaZ_Csp_*	3	CAATTTACCTCT**GAATTC**GGCCACGGCGGGGCCCGGTGCCT
	4	GCGATAAATG**CTCTCGAG**TGGACAATTGCCGACGATGTAGTAGCCGGCGGCCGTCT
*phaZ_Mal_*	5	CTAACTTGCTAC**GAATTC**CGGCCAGACAGATTCCTACACCCTGCCACAG
	6	ATACAGCTAGCA**CTCGAG**GTTACACTGGCCGGCTTCGAAGATGCCGCT
*phaZ_Pst_*	7	CAATTTACGACC**GAATTC**CGGGCAAACCTTCTCCTACACCTCCCCGCAA
	8	GTTATAAACAGT**CTCGAG**GTTGCTGCAGCGTCCGGCCTGGAAATG
*phaZ_Rsp_*	9	CAATTTACCTC**GAATTC**AGCGGTCACCGCCGGGCCCGG
	10	GCGATAAATACA**CTCGAG**CGGGCAGTTGCCGATGACGTAGTAGCCGGC

### 2.2 PhaZ constructs

Genomic DNA was extracted from the PhaZ‐producing strains (RNA/DNA purification kit, Norgen Biotek for *C. testosteroni*, and GeneJET Genomic DNA Purification Kit, ThermoFisher Scientific for other strains). Inserts were obtained by amplifications of the mature *phaZ* genes (without signal peptides)—with primers (5′ to 3′ direction, Table 1c) designed according to the GenBank sequences (Benson et al., 2017) adding restriction sites *Eco*RI and *Xho*I (boldface in Table) through polymerase chain reaction (PCR) (T100 Thermal Cycler, Bio‐Rad) using Phusion High‐Fidelity DNA Polymerase (ThermoFisher Scientific). All PCR products were purified with QIAquick PCR Purification Kit (Qiagen).

Restriction digestions—using *Eco*RI and *Xho*I (NEB)—and ligations—using T4 DNA ligase (ThermoFisher Scientific)—were performed to obtain PhaZs inserts and to incorporate them into the pET‐22b(+) vector (Novagen). This plasmid includes an N‐terminal peIB leader sequence for periplasm localization of the protein, and an optional C‐terminal His‐tag (included in the constructs). Transformation was done chemically with heat shock at 42°C for 30 s or by electroporation with 0.1 cm gap cuvettes at 1.8 kV for 1 s (Gene Pulser, Bio‐Rad), depending on the competent cells required. This was followed by addition of 250 µl of SOC medium (Super Optimal broth with Catabolite repression), incubation for 1 hr at 37°C and 250 rpm, and plating. The constructs were extracted with QIAprep Spin Miniprep Kit (Qiagen) and verified by DNA gel electrophoresis and sequencing (ABI 3730 DNA sequencer; Applied Biosystems). Constructs were inserted in the expression strain *E. coli* Rosetta‐gami B(DE3), and, for PhaZ*_Cte_* and PhaZ*_Mal_*, also in T7 Express *lysY/I^q^ E. coli*.

### 2.3 Induction screening and His‐tag verification

Starter cultures from single colonies of *E. coli* Rosetta‐gami B(DE3) or T7 Express *lysY/I^q^ E. coli* containing the constructs were grown in 5 ml LB with corresponding antibiotics (Table 1b) until reaching an OD_600_ ≈ 0.5 (overnight incubation at ≈ 30°C and 250 rpm is recommended). 15‐ml cultures in LB with antibiotics were inoculated with 1 ml of starter cultures and incubated at 37°C until reaching an OD_600_ ≈ 0.6. Cultures were separated into 3‐ml aliquots, induced with isopropyl‐β‐d‐thiogalactopyranoside (IPTG) (concentrations ranging between 0.01 and 1 mM), and incubated overnight at 15°C (for PhaZ*_Cte_* and PhaZ*_Mal_* incubations at 37°C for 2 or 4 hr were also tested). After incubation, 2 ml of induced cultures were centrifuged (10,000 *g* and 4°C for 10 min) and the pellets were placed at −20°C. B‐PER II Bacterial Protein Extraction Reagent (2×) (ThermoFisher Scientific), supplemented with lysozyme (1 mg/ml, Sigma‐Aldrich) and DNAse I (5 units/ml, ThermoFisher Scientific), was used to obtain soluble fractions (SF) (150 µl/pellet), followed by centrifugation at 21,130 ×*g* and 4°C for 30 min. The same procedure was performed with cells carrying empty pET‐22b(+) vector and with samples before induction.

SF and insoluble fractions (IF) were then characterized by sodium dodecyl sulfate polyacrylamide gel electrophoresis (SDS‐PAGE) analysis, based on the methods described by Laemmli (Laemmli, 1970). Briefly, 2× Laemmli sample buffer (Bio‐Rad) was added to samples and boiled at 100°C for 10 min. Loading volumes in the gel were normalized according to OD_600_ of the samples. A Broad‐Range protein standard ladder (6.5–210 kD) (Bio‐Rad) was used as reference. Samples were run in 12% polyacrylamide gels (Bio‐Rad) for 40 min at constant voltage (200 V). The gels were washed three times with Milli‐Q water for 10 min and stained with PageBlue Protein Staining Solution (ThermoFisher Scientific) for 1 hr under gentle agitation. The gels were then washed with Milli‐Q water. Images of the gels were acquired under UV exposure (AlphaImager EC, Alpha Innotech) or with a regular camera. In the case of PhaZ*_Cte_* and Phaz*_Mal_*, the presence of the His‐tag was verified through Western blot analysis by using mouse anti‐His6 monoclonal antibody, and goat anti‐mouse DyLight 488 secondary antibody (Life Sciences).

### 2.4 Expression and purification of rPhaZs

30 ml of transformed *E. coli* Rosetta‐gami B(DE3) at OD_600_ of 0.5 was added to 1 L LB with antibiotics. Cultures were grown at 37°C for approximately 5 hr, until OD_600_ reached ≈ 0.6. IPTG was added (0.05 and 1 mM for PhaZ*_Mal_*, and 1 mM for all other PhaZs), and the cultures were incubated overnight at 15°C for expression. Cultures were then centrifuged at 10,000 ×*g* and 4°C for 10 min, and the pellets were placed at −20°C. Protein extraction was performed on thawed pellets using 5 ml of B‐PER II mixture with Halt™ Protease Inhibitor Cocktail, EDTA‐Free (100×) (ThermoFisher Scientific) to obtain SF containing PhaZs.

Purification was performed at 4°C using His GraviTrap columns (GE Healthcare). Equilibration was done with 10 ml of B‐PER II before extracted soluble fractions were applied to the column, followed by a wash with 10 ml of binding buffer (50 mM sodium phosphate, 500 mM NaCl, pH 7.4). All three solutions contained 20 mM imidazole. His‐tagged rPhaZs were eluted with 3 ml of elution buffer (20 mM sodium phosphate, 500 mM NaCl, pH 7.4, with 150 mM imidazole for PhaZ*_Mal_* and 500 mM for all other PhaZs). 1‐mL aliquots with 50% glycerol were stored at −20°C. Purified rPhaZs were verified through SDS‐PAGE and quantified with Bradford Protein Assay (microassay procedure, Bio‐Rad) using bovine serum albumin as standard. Amicon Ultra 0.5‐mL filters (Millipore) were used for PhaZ*_C_*_sp_ (molecular weight cut off 30 kDa), PhaZ*_Mal_* (molecular weight cut off 50 kDa), and PhaZ*_R_*_sp_ (molecular weight cut off 30 kDa), which required further purification.

### 2.5 PHB plate rPhaZs activity comparison

PHB degradation assays that are easy to perform and that require little preparation were performed by dispensing 100 µl of soluble fractions in cylindrical wells made in double‐layer mineral medium/agar plates containing PHB (German Culture Collection (DSMZ) medium 474: 20 ml first layer—mineral medium (medium 457, Brunner) with agar (0.016 g/ml)—and 10 ml second layer—mineral medium with agar supplemented with 0.66 ml of sterile PHB suspension). The plates were pierced to produce cylindrical wells for the deposition of samples. Plates were then incubated at 30°C in a temperature‐controlled incubator (Isotemp 500 Series, Fisher Scientific). PHB degradation was assessed by the presence of halos.

The effects of pH and temperature on degradation of PHB by rPhaZs were investigated. Since the PHB plates had pH 7.00, experiments at pH 4.27 and 5.35 were performed using mineral medium with sodium acetate and acetic acid buffer solutions at the desired pH (both solutions at 0.2 M) (Dawson, Elliott, Elliott, & Jones, 1986). The agar and low pH buffer were autoclaved separately. Each rPhaZ was diluted to a concentration of ≈2 µg/ml, and 100 µl was deposited for each condition in duplicates. Plates were incubated at 15°C and 37°C for pH 7.00, and at 37°C for pH 4.27 and 5.35. Pictures were taken over four weeks of incubation. Degradation was assessed by the formation of transparent halos on the PHB plates and measuring the halo diameters (minus well diameter) using ImageJ 1.46r (National Institutes of Health, USA).

## 3 RESULTS AND DISCUSSION

### 3.1 Selection of expression approach

An *E. coli*‐based recombinant protein production system was selected, based on its relative success to produce rPhaZs (Briese et al., 1994; Jendrossek, Frisse, et al., 1995; Jendrossek et al., 1993; Kasuya et al., 2003; Kita et al., 1997; Ohura et al., 1999; Takaku et al., 2006; Takeda et al., 2000; Wang et al., 2012) and the wide commercial offer of vectors and hosts. The sequences of mature PhaZs (based on respective references) were processed using a solubility predictor (PROSO II (Smialowski, Doose, Torkler, Kaufmann, & Frishman., 2012; Smialowski et al., 2011)), and the theoretical isoelectric points (pI) and molecular weights (Mw) were calculated using the Compute pI/Mw tool from the ExPASy Bioinformatics Resources Portal (SIB) (Artimo et al., 2012; Gasteiger et al., 2005; SIB Swiss Institute of Bioinformatics n.d.) (Table 2). Since PhaZ*_Cte_* was classified as insoluble (predicted solubility score 0.503), and the other PhaZs had scores (0.657–0.765) near the PROSO II threshold for solubility of 0.6, insolubility was considered a potential drawback for production of rPhaZs. To overcome potential insolubility issues, induction was performed overnight at 15°C. In addition, extracellular PhaZs are known to be sensitive to dithiothreitol (DTT), suggesting they likely form disulfide bonds (Jendrossek & Handrick, 2002); in fact, the DiANNA 1.1 web server tool predicted several disulfide bonds for the mature PhaZs used in this study (Ferrè & Clote, 2005a,b, 2006). The plasmid pET‐22b(+)—previously used to express fusion proteins of the substrate‐binding domain of PhaZ*_Pst_* (Park et al., 2005), and PhaZs from *P. mendocina* DSWY0601[19] and *Bacillus* sp. NRRL B‐14911 (Ma, Lin, Chen, Chen, & Shaw, 2011)—containing the N‐terminal peIB leader sequence for periplasm localization (a more favorable environment for disulfide bond formation) was selected. In addition, we selected the *E. coli* expression strain Rosetta‐gami B(DE3) and, as an alternative, T7 Express *lysY/I^q^* (allowing cloning and expression of toxic genes through tight control of expression by *lacl^q^* and of T7 RNA Polymerase by lysozyme). Both strains are BL21 derivates designed to aid in expression of proteins that contain disulfide bonds and suitable for expression under T7 promoter. Furthermore, Rosetta‐gami B(DE3) *E. coli* contains the plasmid pRARE that supplies tRNAs for five rare codons (three present in PhaZ*_Cte_*, one in PhaZ*_C_*_sp_, and two in PhaZ*_Mal_*, PhaZ*_Pst_*, and PhaZ*_R_*_sp_).

**Table 2:** PhaZs properties, sequence analyses, qualitative expression, and activity. Relative activity was tested on PHB plates where rPhaZs were deposited and displayed degradation as transparent halos

Strain	PhaZ	PhaZ GenBank, reference	Theoretical mature PhaZ pI/Mw (kDa)	Predicted solubility score, class	Predicted disulfide bonds	Soluble expression level^a^	Relative activity on PHB plates^a^, ^b^
*Comamonas testosteroni* 31A	PhaZ*_Cte_*	U16275.1 (Jendrossek, Backhaus, et al., 1995)	7.64/50.6	0.503 insoluble	5	++	1. pH 7.0, 37°C: + 2. pH 7.0, 15°C: − 3. pH 5.4, 37°C: ±‐
*Cupriavidus* sp. T1	PhaZ*_C_*_sp_	J04223.2 (Saito et al., 1989)	6.03/46.9	0.735 soluble	3	–	1. pH 7.0, 37°C: + 2. pH 7.0, 15°C: ±− 3. pH 5.4, 37°C: ±
*Marinobacter algicola* DG893	PhaZ*_Mal_*	ABCP01000004.1 and EDM48791.1 (Green et al., 2006, 2007a,b)	4.33/58.7	0.765 soluble	6	–	1. pH 7.0, 37°C: + 2. pH 7.0, 15°C: ± 3. pH 5.4, 37°C: ‐‐
*Pseudomonas stutzeri*	PhaZ*_Pst_*	AB012225.1 (Ohura et al., 1999)	5.32/57.5	0.657 soluble	6	+	1. pH 7.0, 37°C: + 2. pH 7.0, 15°C: − 3. pH 5.4, 37°C: ‐‐‐
*Ralstonia* sp.	PhaZ*_R_*_sp_	D25315.1 (Yukawa, Uchida, Kohama, & Kurusu, 1994)	6.03/47.6	0.758 soluble	3	−	1. pH 7.0, 37°C: + 2. pH 7.0, 15°C: ‐ 3. pH 5.4, 37°C: ±

a PhaZ expression levels on SDS‐PAGE and activity on PHB plates: very high (++), high (+), medium (±), low (‐), very low (‐‐), and inactive/insoluble (‐‐‐).

b Activity was monitored over 1 week of incubation.

Successful pET‐22b(+)‐PhaZs constructs were obtained for all cloned PhaZs. Sequencing of mature *phaZ* inserts revealed the sequences of PhaZ*_Mal_* and PhaZ*_Pst_* were the same as in the Genbank registers, while some changes were found for PhaZ*_Cte_*, PhaZ*_C_*_sp_, and PhaZ*_R_*_sp_, that resulted in 3 (T42I, T457N, and A458G—sequencing from PCR of genomic DNA extracted from 4 colonies of *C. testosteroni*), 1 (N236T—sequencing from 2 constructs), and 7 (A169G, I170T, R172T, W173S, K174Q, N285T, and A318G—sequencing from 2 constructs and last 2 changes confirmed by sequencing of another PCR of genomic DNA) amino acid changes, respectively, with position numbering starting from the first amino acid of each mature peptide (as defined by (Jendrossek, Backhaus, & Andermann, 1995) for PhaZ*_Cte_*, (Saito et al., 1989) for PhaZ*_C_*_sp_, and (Shiraki, Shimada, Tatsumichi, & Saito, 1995) for PhaZ*_R_*_sp_). This was likely due to variations in the taxonomic strains (Dijkshoorn, Ursing, & Ursing, 2000) since their deposition.

### 3.2 Expression and purification of rPhaZs

Conditions for induction were established with the expression strain *E. coli* Rosetta‐gami B(DE3). SDS‐PAGE showed that PhaZ*_Cte_* and PhaZ*_Pst_* were present in both SF and IF for inductions with 1 mM IPTG at 15°C. Decreasing the IPTG concentration reduced insoluble rPhaZs, but the maximum expression in SF was observed at 1 mM. PhaZ*_C_*_sp_ expression was limited in both SF and IF, while PhaZ*_R_*_sp_ showed higher accumulation in the IF; the same phenomenon was observed for PhaZ*_Mal_*. When inductions were carried at 37°C, PhaZ*_Cte_* and PhaZ*_Mal_* were only present in the IFs.

**Figure 1:**
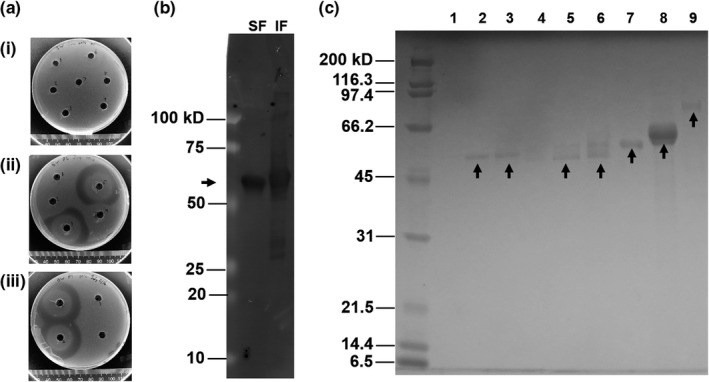
Examples of assays for production of recombinant PhaZs. (a) Induction screening of PhaZ*_Mal_* on PHB plates (SFs, 30°C, 7 days incubation): (i) and (ii) T7 Express *lysY/I^q^ E. coli* (induced with IPTG 0.05–1 mM, at 37°C for 2 hr and 15°C overnight, respectively, in duplicates (R1, R2)), wells (i) 1: R1, 1 mM, 37°C, 2: R1, 1 mM, 15°C, 3: R2, 1 mM, 37°C, 4: R2, 1 mM, 15°C, 5: R1, 0.4 mM, 37°C, 6: R1, 0.4 mM, 15°C, 7: R2, 0.4 mM, 37°C, and (ii) 1: R2, 0.4 mM, 15°C, 2: R1, 0.05 mM, 37°C, 3: R1, 0.05 mM, 15°C, 4: R2, 0.05 mM, 37°C, 5: R2, 0.05 mM, 15°C; and (iii) *E. coli* Rosetta‐gami B(DE3) (induced with IPTG 0.01–1 mM, at 15°C overnight), wells 1: 1 mM, 2:0.4 mM, 3:0.05 mM, 4:0.01 mM. (b) Western blot of SF and IF of rPhaZ*_Cte_* containing a His‐tag (induced with 1 mM IPTG, at 15°C overnight). (c) Purified rPhaZs: lanes 1 to 9 are, respectively, as follows: PhaZ*_C_*_sp_ F, PhaZ*_C_*_sp_ C, PhaZ*_C_*_sp_, PhaZ*_R_*_sp_ F, PhaZ*_R_*_sp_ C, PhaZ*_R_*_sp_, PhaZ*_Cte_*, PhaZ*_Pst_*, and PhaZ*_Mal_* C, where F: filtrate and C: concentrate from size exclusion columns

Activity was verified for SFs in PHB plates incubated at 30°C (example shown in Figure 1a for PhaZ*_Mal_*, for which clear zones were only observed for soluble fractions from cultures induced at 15°C with 0.05 mM IPTG for T7 Express *lysY/I^q^ E. coli*, and 0.4 or 1 mM IPTG for *E. coli* Rosetta‐gami B(DE3)). T7 Express *lysY/I^q^ E. coli* was tested with PhaZ*_Mal_* to see if low expression could be due to gene toxicity, but expression was not improved and active enzymes could not be produced with induction above 0.05 mM IPTG even at 15°C. The ring effect observed in some samples was likely due to self‐inhibition at high concentrations of rPhaZ (Martínez‐Tobón, Gul, Elias, & Sauvageau, 2018; Mukai, Yamada, & Doi, 1993; Uefuji, Kasuya, & Doi, 1997). The combined SDS‐PAGE/PHB plate analysis is important because rPhaZs at low expression levels were not always observed by SDS‐PAGE—the PHB plate assay helps confirm the presence of active rPhaZs in the SFs. For the other rPhaZs, SFs only showed activity when cultures were induced at 15°C. The favored induction conditions were determined to be 15°C with 1 mM IPTG for PhaZ*_Cte_*, PhaZ*_C_*_sp_, PhaZ*_R_*_sp_, and PhaZ*_Pst_* and 0.05 mM IPTG for PhaZ*_Mal_*.

Removing the native signal peptide from the PhaZ sequence is a key step in diminishing the formation of inclusion bodies and avoiding completely insoluble PhaZs when using pET‐22b(+) or plasmids that add signal sequences. Induction at higher IPTG concentrations and addition of ethanol in the induction stage (Chhetri, Kalita, & Tripathi, 2015) did not lead to improved expression.

The presence of the C‐terminal His‐tag was verified through Western blot for SF and IF of PhaZ*_Cte_* and PhaZ*_Mal_* before proceeding to large‐scale inductions for purification. An example is shown in Figure 1b, c for the SF and IF of PhaZ*_Cte_* induced at 15°C with 1 mM IPTG. After 1‐L inductions, all rPhaZs could be separated through simple His‐tag based purification (as confirmed by SDS‐PAGE in Figure 1c, d, e). Relative expression levels in the SF of each PhaZ were qualitatively classified as very high for PhaZ*_Cte_*, high for PhaZ*_Pst_*, low for PhaZ*_Mal_* and PhaZ*_R_*_sp_, and very low for PhaZ*_C_*_sp_ (Table 2). Purification, which was especially challenging for PhaZ*_Mal_*, could be further improved using a combination of strategies, including doing the equilibration, sample application, and wash steps with solutions containing 50 mM imidazole—this improved purity of PhaZ*_Cte_*, PhaZ*_Mal_*, and PhaZ*_Rsp_*, at the expense of recovery—adding an elution step with 150 mM imidazole instead of 500 mM for PhaZ*_Mal_*, and using size exclusion columns.

### 3.3 Comparison of rPhaZ activity

While PHB plates have been mostly used to screen for PHB‐degrading bacteria (Jendrossek, 2005), activity from expressed PhaZs has also been estimated by clear zones on glass slides covered by PHB‐agar mix (Briese et al., 1994; Jendrossek et al., 1993) (this test is limited to short‐term incubations due to agar drying—unless a humidity chamber is used for incubation—but is advantageous for preliminary assessment and when only small volumes of sample are available).

**Figure 2:**
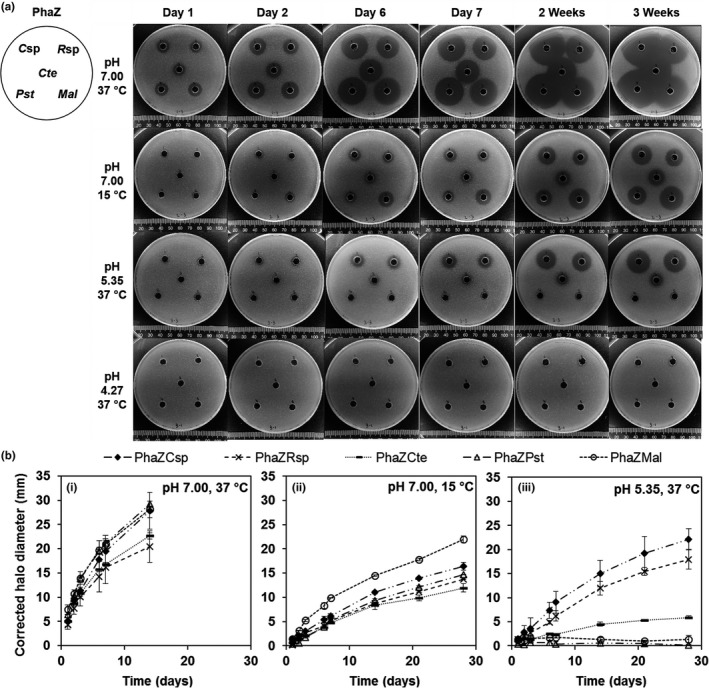
Degradation activity of rPhaZs on PHB plates. rPhaZs concentration was ≈2 µg/ml. (a) Degradation as a function of time under different temperatures and pH values. Halos indicate degradation. (b) Degradation halos diameter (corrected for well diameter, mean ± *SD*, *n* = 2). (i) pH 7.00, 37°C; (ii) pH 7.00, 15°C; and (iii) pH 5.35, 37°C. No halos were observed at pH 4.27, 37°C

In this study, an easy‐to‐use method using PHB plates was used to compare PhaZ activity at various pH and temperatures and provided semiquantitative assessments of activity based on the diameter of degradation halos formed (Figure 2). At 37°C, degradation was observed on the first day of incubation for all rPhaZs tested, while longer incubation periods were required at 15°C. PhaZ*_Mal_* showed the highest activity at 15°C (halo observed after 1 day) compared with the other rPhaZs (halos observed after 6 days), which could be explained by its marine origin (Green, Bowman, Smith, Gutierrez, & Bolch, 2006). All enzymes were rendered inactive at pH 4.3 (no halos discernable) but PhaZ*_C_*_sp_ and PhaZ*_R_*_sp_ retained significant activity at pH 5.4, which can render these enzymes useful for applications under conditions below neutral or alkaline pH. This is important as most PhaZs identified to date display optimal activity at higher pH and inactivity at low pH. Also, this is consistent with their broad pH working ranges (PhaZ*_C_*_sp_ is stable when stored at pH 5.0–8.0 (Kasuya, Inoue, Yamada, & Doi, 1995) with optimum activity at pH 7.5 (Tanio et al., 1982), and the optimum pH range of PhaZ*_R_*_sp_ is 5.0–6.0 (Yamada, Mukai, & Doi, 1993)).

These results could be confirmed and semiquantified by comparing the rate of change of the degradation halos under the different conditions tested (Figure 2). For example, similar degradation rates were observed for all rPhaZs at 37°C and pH 7.0, but PhaZ*_Mal_* had a noticeably greater rate at 15°C and pH 7.0 (leading to ≈33% more degradation after 28 hr). Such methods represent powerful tools for screening recombinant and engineered PhaZs, as was demonstrated by Hiraishi et al. who used LB plates containing PHB granules, IPTG, and antibiotics to evaluate clear zone activity of PhaZ mutants (Hiraishi et al., 2006).

## 4 CONCLUSIONS

This study presents a streamlined platform for the rapid production of rPhaZs. Five active PHB‐degrading extracellular PhaZs (PhaZ*_Cte_*, PhaZ*_Pst_*, PhaZ*_C_*_sp_ PhaZ*_R_*_sp_, and PhaZ*_Mal_*), originating from bacteria from diverse environments, were successfully produced in the SF of Rosetta‐gami B(DE3) *E. coli*. An important aspect of the method requires the removal of the native signal peptide sequence of PhaZ to avoid production of insoluble proteins and inactive enzymes. Expression levels and purity varied for each enzyme—PhaZ*_Cte_* and PhaZ*_Pst_* saw highest expression—but they could all be recovered and retained activity. In addition, degradation activity could easily be assessed by determining the diameter of degradation halos in PHB plates. This assay can be done in parallel for the initial screening of PhaZs and conditions for diverse applications. Both the rPhaZ production platform and the modified PHB plate assay are versatile and reliable, and could be employed with other PhaZs reported in the literature or novel ones to be discovered or synthesized.

## CONFLICT OF INTEREST

None declared.

## AUTHOR CONTRIBUTION

Diana Martínez Tobón contributed equally to conceptualization, took the lead in formal analysis, investigation, methodology, writing–original draft, and writing–review and editing. Brennan Waters supported in investigation and methodology. Anastasia Elias contributed equally to conceptualization, funding acquisition, project administration, supervision, and writing–review and editing, and supported in methodology. Dominic Sauvageau contributed equally to conceptualization, funding acquisition, project administration, supervision, writing–review and editing, and supported in formal analysis, methodology, and writing–original draft.

## ETHICS STATEMENT

None required.

## Data Availability

All data generated in this study are available from the corresponding author upon reasonable request.

## References

[mbo31001-bib-0001] Anbukarasu, P. , Sauvageau, D. , & Elias, A. L. (2017). Time‐temperature indicator based on enzymatic degradation of dye‐loaded polyhydroxybutyrate. Biotechnology Journal, 12(9), 1700050. 10.1002/biot.201700050 28805014

[mbo31001-bib-0002] Artimo, P. , Jonnalagedda, M. , Arnold, K. , Baratin, D. , Csardi, G. , de Castro, E. , … Stockinger, H. (2012). ExPASy: SIB bioinformatics resource portal. Nucleic Acids Research, 40, W597–W603. 10.1093/nar/gks400 22661580 PMC3394269

[mbo31001-bib-0003] Benson, D. , Cavanaugh, A. M. , Clark, K. , Karsch‐Mizrachi, I. , Lipman, D. J. , Ostell, J. , & Sayers, E. W. (2017). GenBank. Nucleic Acids Research, 45(D1), D37–D42. 10.1093/nar/gkw1070 27899564 PMC5210553

[mbo31001-bib-0004] Braaz, R. , Handrick, R. , & Jendrossek, D. (2003). Identification and characterisation of the catalytic triad of the alkaliphilic thermotolerant PHA depolymerase PhaZ7 of *Paucimonas lemoignei* . FEMS Microbiology Letters, 224(1), 107. 12855176 10.1016/S0378-1097(03)00425-7

[mbo31001-bib-0005] Briese, B. H. , Schmidt, B. , & Jendrossek, D. (1994). *Pseudomonas lemoignei* has five poly(hydroxyalkanoic Acid) (PHA) depolymerase genes: A comparative study of bacterial and eukaryotic PHA depolymerases. Journal of Environmental Polymer Degradation, 2(2), 75–87. 10.1007/BF02074776

[mbo31001-bib-0006] Chhetri, G. , Kalita, P. , & Tripathi, T. (2015). An efficient protocol to enhance recombinant protein expression using ethanol in *Escherichia coli* . MethodsX 2, 385–391. 10.1016/j.mex.2015.09.005 26629417 PMC4635407

[mbo31001-bib-0007] Dawson, R. , Elliott, D. C. , Elliott, W. H. , & Jones, K. M. (1986). Data for biochemical research. Oxford, UK: Oxford Science Publications.

[mbo31001-bib-0008] Dijkshoorn, L. , Ursing, B. M. , & Ursing, J. B. (2000). Strain, clone and species: Comments on three basic concepts of bacteriology. Journal of Medical Microbiology, 49(5), 397–401. 10.1099/0022-1317-49-5-397 10798550

[mbo31001-bib-0009] Elias, A. , Sauvageau, D. , Storms, Z. , Wang, C. , Anbukarasu, P. , & Martinez‐Tobon, D. (2018). Bacteriophage‐based biosensor for microbial detection. US9921219B2.

[mbo31001-bib-0010] Ferrè, F. , & Clote, P. (2005a). DiANNA: A web server for disulfide connectivity prediction. Nucleic Acids Research, 33, W230–W232. 10.1093/nar/gki412 15980459 PMC1160173

[mbo31001-bib-0011] Ferrè, F. , & Clote, P. (2005b). Disulfide connectivity prediction using secondary structure information and diresidue frequencies. Bioinformatics, 21, 2336–2346. 10.1093/bioinformatics/bti328 15741247

[mbo31001-bib-0012] Ferrè, F. , & Clote, P. (2006). DiANNA 1.1: An extension of the DiANNA web server for ternary cysteine classification. Nucleic Acids Research, 34, W182–W185. 10.1093/nar/gkl189 16844987 PMC1538812

[mbo31001-bib-0013] Gasteiger, E. , Hoogland, C. , Gattiker, A. , Duvaud, S. , Wilkins, M. R. , Appel, R. D. , & Bairoch, A. (2005). Protein identification and analysis tools on the ExPASy server In Walker J. M., ed. The proteomics protocols handbook (pp. 571–607). Humana Press 10.1385/1-59259-890-0:571

[mbo31001-bib-0014] Geyer, R. , Jambeck, J. R. , & Law, K. L. (2017). Production, use, and fate of all plastics ever made. Science Advances, 3(7), e1700782 10.1126/sciadv.1700782 28776036 PMC5517107

[mbo31001-bib-0015] Green, D. H. , Bowman, J. P. , Smith, E. A. , Gutierrez, T. , & Bolch, C. J. S. (2006). *Marinobacter algicola* sp. nov., isolated from laboratory cultures of paralytic shellfish toxin‐producing dinoflagellates. International Journal of Systematic and Evolutionary Microbiology, 56(3), 523–527. 10.1099/ijs.0.63447-0 16514021

[mbo31001-bib-0016] Green, D. , Ferriera, S. , Johnson, J. , Kravitz, S. , Beeson, K. , Sutton, G. , … Venter, J. C. (2007a). *Marinobacter algicola* DG893 1103407001893, whole genome shotgun sequence. Direct Submission. GenBank: ABCP01000004.1.

[mbo31001-bib-0017] Green, D. , Ferriera, S. , Johnson, J. , Kravitz, S. , Beeson, K. , Sutton, G. , … Venter, J. C. (2007b). poly(3‐hydroxybutyrate) depolymerase [*Marinobacter algicola* DG893]. Direct Submission. GenBank: EDM48791.1.

[mbo31001-bib-0018] Hiraishi, T. , Hirahara, Y. , Doi, Y. , Maeda, M. , & Taguchi, S. (2006). Effects of mutations in the substrate‐binding domain of Poly[(R)‐3‐hydroxybutyrate] (PHB) depolymerase from *Ralstonia pickettii* T1 on PHB degradation. Applied and Environmental Microbiology, 72(11), 7331–7338. 10.1128/AEM.01187-06 16963553 PMC1636158

[mbo31001-bib-0019] Hiraishi, T. , Komiya, N. , Matsumoto, N. , Abe, H. , Fujita, M. , & Maeda, M. (2009). Degradation and adsorption characteristics of PHB depolymerase as revealed by kinetics of mutant enzymes with amino acid substitution in substrate‐binding domain. Biomacromolecules, 11, 113–119. 10.1021/bm900967a 20058938

[mbo31001-bib-0020] JCM (2019). *Cupria vidus* sp. Retrieved from https://www.jcm.riken.jp/cgi-bin/jcm/jcm_number?JCM=10169 (Accessed 25 November 2019).

[mbo31001-bib-0021] Jendrossek, D. (2005). Extracellular polyhydroxyalkanoate depolymerases: The key enzymes of PHA degradation In Steinbüchel A. (Ed.), Biopolymers online. Wiley.

[mbo31001-bib-0022] Jendrossek, D. , Backhaus, M. , & Andermann, M. (1995). Characterization of the extracellular poly(3‐hydroxybutyrate) depolymerase of *Comamonas* sp. and of its structural gene. Canadian Journal of Microbiology, 41(13), 160–169. 7606660 10.1139/m95-183

[mbo31001-bib-0023] Jendrossek, D. , Frisse, A. , Behrends, A. , Andermann, M. , Kratzin, H. D. , Stanislawski, T. , & Schlegel, H. G. (1995). Biochemical and molecular characterization of the *Pseudomonas lemoignei* polyhydroxyalkanoate depolymerase system. Journal of Bacteriology, 177(3), 596–607. 10.1128/JB.177.3.596-607.1995 7836292 PMC176633

[mbo31001-bib-0024] Jendrossek, D. , & Handrick, R. (2002). Microbial degradation of polyhydroxyalkanoates*. Annual Review of Microbiology, 56(1), 403–432. 10.1146/annurev.micro.56.012302.160838 12213937

[mbo31001-bib-0025] Jendrossek, D. , Hermawan, S. , Subedi, B. , & Papageorgiou, A. C. (2013). Biochemical analysis and structure determination of *Paucimonas lemoignei* poly(3‐hydroxybutyrate) (PHB) depolymerase PhaZ7 muteins reveal the PHB binding site and details of substrate–enzyme interactions. Molecular Microbiology, 90(3), 649–664. 24007310 10.1111/mmi.12391

[mbo31001-bib-0026] Jendrossek, D. , Müller, B. , & Schlegel, H. G. (1993). Cloning and characterization of the poly(hydroxyalkanoic acid)‐depolymerase gene locus, *phaZ1*, of *Pseudomonas lemoignei* and its gene product. European Journal of Biochemistry, 218(2), 701–710. 10.1111/j.1432-1033.1993.tb18424.x 8269961

[mbo31001-bib-0027] Kasuya, K.‐I. , Inoue, Y. , Yamada, K. , & Doi, Y. (1995). Kinetics of surface hydrolysis of poly[(R)‐3‐hydroxybutyrate] film by PHB depolymerase from *Alcaligenes faecalis* T1. Polymer Degradation and Stability, 48(1), 167–174. 10.1016/0141-3910(95)00026-I

[mbo31001-bib-0028] Kasuya, K.‐I. , Takano, T. , Tezuka, Y. , Hsieh, W. C. , Mitomo, H. , & Doi., Y. (2003). Cloning, expression and characterization of a poly(3‐hydroxybutyrate) depolymerase from *Marinobacter* sp. NK‐1. International Journal of Biological Macromolecules, 33(4–5), 221–226. 10.1016/j.ijbiomac.2003.08.006 14607367

[mbo31001-bib-0029] Kita, K. , Mashiba, S. I. , Nagita, M. , Ishimaru, K. , Okamoto, K. , Yanase, H. , & Nobuo, K. (1997). Cloning of poly(3‐hydroxybutyrate) depolymerase from a marine bacterium, *Alcaligenes faecalis* AE122, and characterization of its gene product. Biochimica Et Biophysica Acta, 1352, 113–122. 10.1016/S0167-4781(97)00011-0 9177489

[mbo31001-bib-0030] Knoll, M. , Hamm, T. M. , Wagner, F. , Martinez, V. , & Pleiss, J. (2009). The PHA depolymerase engineering database: a systematic analysis tool for the diverse family of polyhydroxyalkanoate (PHA) depolymerases. BMC Bioinformatics, 10(Database), 89. 19296857 10.1186/1471-2105-10-89 PMC2666664

[mbo31001-bib-0031] Laemmli, U. K. (1970). Cleavage of structural proteins during the assembly of the head of bacteriophage T4. Nature, 227, 680–685. 5432063 10.1038/227680a0

[mbo31001-bib-0032] Lee, M. C. , Liu, E. J. , Yang, C. H. , Hsiao, L. J. , Wu, T. M. , & Li, S. Y. (2018). Co‐expression of ORF_*Cma*_ with PHB depolymerase (PhaZ_*Cma*_) in *Escherichia coli* induces efficient whole‐cell biodegradation of polyesters. Biotechnology Journal, 13, 1700560 10.1002/biot.201700560 29337429

[mbo31001-bib-0033] Ma, W.‐T. , Lin, J. H. , Chen, H. J. , Chen, S. Y. , & Shaw, G. C. (2011). Identification and characterization of a novel class of extracellular poly(3‐Hydroxybutyrate) depolymerase from *Bacillus* sp. strain NRRL B‐14911. Applied and Environmental Microbiology, 77, 7924–7932. 10.1128/AEM.06069-11 21948827 PMC3208993

[mbo31001-bib-0034] Martínez‐Tobón, D. I. , Gul, M. , Elias, A. L. , & Sauvageau, D. (2018). Polyhydroxybutyrate (PHB) biodegradation using bacterial strains with demonstrated and predicted PHB depolymerase activity. Applied Microbiology and Biotechnology, 102(18), 8049–8067. 10.1007/s00253-018-9153-8 29951858

[mbo31001-bib-0035] Mukai, K. , Yamada, K. , & Doi, Y. (1993). Kinetics and mechanism of heterogeneous hydrolysis of poly [(R)‐3‐hydroxybutyrate] film by PHA depolymerases. International Journal of Biological Macromolecules, 15, 361–366. 10.1016/0141-8130(93)90054-P 8110658

[mbo31001-bib-0036] Ohura, T. , Kasuya, K.‐I. , & Doi, Y. (1999). Cloning and characterization of the polyhydroxybutyrate depolymerase gene of *Pseudomonas stutzeri* and analysis of the function of substrate‐binding domains. Applied and Environmental Microbiology, 65(1), 189–197. 10.1128/AEM.65.1.189-197.1999 9872779 PMC91002

[mbo31001-bib-0037] Oshida, Y. , Kitamura, K. , Iida, T. , & Ohkuma, M. (2015). *Cupriavidus* sp. JCM 10169 gene for 16S ribosomal RNA, partial sequence. Direct Submission. GenBank: LC107433.1.

[mbo31001-bib-0038] Park, J. P. , Lee, K. B. , Lee, S. J. , Park, T. J. , Kim, M. G. , Chung, B. H. , Lee, Z. W. , Choi, I. S. , & Lee, S. Y. (2005). Micropatterning proteins on polyhydroxyalkanoate substrates by using the substrate binding domain as a fusion partner. Biotechnology & Bioengineering, 92, 160–165. 16028291 10.1002/bit.20581

[mbo31001-bib-0039] Roohi , Bano, K. , Kuddus, M. , Zaheer, M. R. , Zia, Q. , Khan, M. F. , Ashraf, G. M. , Gupta, A. , & Aliev, G. (2017). Microbial enzymatic degradation of biodegradable plastics. Current Pharmaceutical Biotechnology, 18, 429–440. 10.2174/1389201018666170523165742 28545359

[mbo31001-bib-0040] Rosano, G. L. , & Ceccarelli, E. A. (2014). Recombinant protein expression in *Escherichia coli*: Advances and challenges. Frontiers in Microbiology, 5, 172 10.3389/fmicb.2014.00172 24860555 PMC4029002

[mbo31001-bib-0041] Saito, T. , Suzuki, K. , Yamamoto, J. , Fukui, T. , Miwa, K. , Tomita, K. , … Ishikawa, K. (1989). Cloning, nucleotide sequence, and expression in *Escherichia coli* of the gene for poly(3‐hydroxybutyrate) depolymerase from *Alcaligenes faecalis* . Journal of Bacteriology, 171(1), 184–189. 10.1128/JB.171.1.184-189.1989 2644188 PMC209571

[mbo31001-bib-0042] Shiraki, M. , Shimada, T. , Tatsumichi, M. , & Saito, T. (1995). Purification and characterization of extracellular poly(3‐hydroxybutyrate) depolymerases. Journal of Environmental Polymer Degradation, 3(1), 13–21. 10.1007/BF02067789

[mbo31001-bib-0043] Smialowski, P. , Doose, G. , Torkler, P. , Kaufmann, S. , & Frishman, D. (2012). PROSO II–a new method for protein solubility prediction. FEBS, 279, 2192–2200. 10.1111/j.1742-4658.2012.08603.x 22536855

[mbo31001-bib-0044] Smialowski, P. , Doose, G. , Torkler, P. , Kaufmann, S. & Frishman, D. (2011). Expropriator Web server: PROSO II. http://mbiljj45.bio.med.uni-muenchen.de:8888/prosoII/prosoII.seam (Accessed 7 November 2018).

[mbo31001-bib-0045] SIB Swiss Institute of Bioinformatics, Compute pI/Mw. https://web.expasy.org/compute_pi/ (Accessed 7 November 2018).

[mbo31001-bib-0046] Takaku, H. , Kimoto, A. , Kodaira, S. , Nashimoto, M. , & Takagi, M. (2006). Isolation of a Gram‐positive poly (3‐hydroxybutyrate)(PHB)‐degrading bacterium from compost, and cloning and characterization of a gene encoding PHB depolymerase of *Bacillus megaterium* N‐18‐25‐9. FEMS Microbiology Letters, 264, 152–159. 17064368 10.1111/j.1574-6968.2006.00448.x

[mbo31001-bib-0047] Takeda, M. , Kitashima, K. , Adachi, K. , Hanaoka, Y. , Suzuki, I. , & Koizumi, J.‐I. (2000). Cloning and expression of the gene encoding thermostable poly(3‐hydroxybutyrate) depolymerase. Journal of Bioscience and Bioengineering, 90(4), 416–421. 10.1016/S1389-1723(01)80011-6 16232882

[mbo31001-bib-0048] Tan, L.‐T. , Hiraishi, T. , Sudesh, K. , & Maeda, M. (2013). Directed evolution of poly[(R)‐3‐hydroxybutyrate] depolymerase using cell surface display system: Functional importance of asparagine at position 285. Applied Microbiology and Biotechnology, 97(11), 4859–4871. 22940802 10.1007/s00253-012-4366-8

[mbo31001-bib-0049] Tan, L.‐T. , Hiraishi, T. , Sudesh, K. , & Maeda, M. (2014). Effects of mutation at position 285 of *Ralstonia pickettii* T1 poly[(R)‐3‐hydroxybutyrate] depolymerase on its activities. Applied Microbiology and Biotechnology, 98(16), 7061–7068. 24676749 10.1007/s00253-014-5660-4

[mbo31001-bib-0050] Tanio, T. , Fukui, T. , Shirakura, Y. , Saito, T. , Tomita, K. , Kaiho, T. , & Masamune, S. (1982). An extracellular poly(3‐hydroxybutyrate) depolymerase from *Alcaligenes faecalis* . European Journal of Biochemistry, 124(1), 71–77. 10.1111/j.1432-1033.1982.tb05907.x 7084231

[mbo31001-bib-0051] Uefuji, M. , Kasuya, K.‐I. , & Doi, Y. (1997). Enzymatic degradation of poly[(R)3‐hydroxybutyrate]: Secretion and properties of PHB depolymerase from Pseudomonas stutzeri. Polymer Degradation and Stability, 58(3), 275–281. 10.1016/S0141-3910(97)00058-X

[mbo31001-bib-0052] Volova, T. G. (2004). Polyhydroxyalkanoates–plastic materials of the 21st century. New York: Nova Science Publishers.

[mbo31001-bib-0053] Wang, Y. , Li, F. , Wang, Z. , Liu, D. , Xia, H. , Liu, L. , & Chen, S. (2012). Purification and properties of an extracellular polyhydroxybutyrate depolymerase from *Pseudomonas mendocina* DSWY0601. Chemical Research in Chinese Universities, 28(3), 459–463.

[mbo31001-bib-0054] Yamada, K. , Mukai, K. , & Doi, Y. (1993). Enzymatic degradation of poly(hydroxyalkanoates) by *Pseudomonas pickettii* . International Journal of Biological Macromolecules, 15(4), 215–220. 10.1016/0141-8130(93)90040-S 8373740

[mbo31001-bib-0055] Yukawa, H. , Uchida, Y. , Kohama, K. , & Kurusu, Y. (1994). Monitoring of polymer biodegradabilities in the environment by a DNA probe method In Doi Y., & Fukuda K. (Eds.), Biodegradable plastics and polymers (pp. 65–76). Elsevier Science.

